# A new species of *Mariauxiella* de Chambrier & Rego, 1995 (Cestoda: Proteocephalidae) infecting the driftwood catfish *Ageneiosus militaris* Valenciennes (Siluriformes: Auchenipteridae) in Brazil

**DOI:** 10.1007/s11230-026-10298-4

**Published:** 2026-08-11

**Authors:** Philippe Vieira Alves, Hugo Lago Rocha, Reinaldo José da Silva

**Affiliations:** Instituto de Biociências, Departamento de Biodiversidade e Bioestatística (Setor de Parasitologia), Universidade Estadual Paulista “Júlio de Mesquita Filho” (UNESP), Rua Prof. Dr. Antônio Celso Wagner Zanin, s/n, Botucatu, São Paulo 18616-689 Brazil

## Abstract

The tapeworm genus *Mariauxiella* (Onchoproteocephalidea, Proteocephalidae) was recently revised and its species composition reappraised; however, its actual species diversity remains incompletely understood. In the present study, an integrative taxonomic approach combining morphological, ultrastructural, and molecular phylogenetic analyses was applied to proteocephalid tapeworms collected from the driftwood catfish *Ageneiosus militaris* (Siluriformes: Auchenipteridae) in the Upper Paraná River basin, Brazil. The evidence obtained supports the proposal of *Mariauxiella saltogrande* **n. sp.**, the third species assigned to the genus. The new species can be differentiated from the most similar species, *Mariauxiella brevifilis*, by its body size, proglottis width, scolex shape, and the arrangement of the osmoregulatory canals in the proglottides. Phylogenetic analyses based on the partial large subunit nuclear ribosomal RNA (RNA28S) and the complete mitochondrial cytochrome *c* oxidase subunit I (MT-CO1) sequence data strongly supported the monophyly of *Mariauxiella* and recovered *Mariauxiella saltogrande* n. sp. as sister to *M. brevifilis*. The discovery of this new species expands the known diversity of *Mariauxiella* and provides further evidence for the usefulness of integrative taxonomy in resolving species boundaries and evolutionary relationships among Neotropical proteocephalids.

**Supplementary Information:**

The online version contains supplementary material available at https://doi.org/10.1007/s11230-026-10298-4.

## Introduction

Neotropical proteocephalids (Onchoproteocephalidea, Proteocephalidae) represent the largest radiation of adult tapeworms infecting bony fishes worldwide (Scholz & Kuchta, 2022). However, their true diversity is likely much higher than currently recognized, with around 90% of the total number of species yet to be discovered in South American freshwater fishes (Scholz & Kuchta, 2022). This estimate is plausible because most representatives of the main group of teleosts harboring adult cestodes, i.e., Neotropical catfishes (Siluriformes), have never been scrutinized for tapeworm infections (Alves et al., 2017a; Scholz & Kuchta, 2022).

Beyond the still poorly known proteocephalid diversity in South American fishes, another limitation is that most genera and higher-level taxa were established solely based on morphological traits (Rego, 1994; de Chambrier et al., 2017). These characteristics have failed to reflect the evolutionary relationships within the group, as revealed by molecular phylogenetic analyses (de Chambrier et al., 2015a, 2017), and a natural classification is still pending for many genera of Proteocephalidae (Alves et al., 2025, 2026). Relying on combined morphological and molecular evidence, Alves et al. (2021b) addressed this issue and proposed a taxonomic rearrangement of three Neotropical genera infecting freshwater fishes: *Ageneiella* de Chambrier & Vaucher, 1999, *Manaosia* Woodland, 1935, and *Mariauxiella* de Chambrier & Rego, 1995. As a result, *Manaosia* now comprises *Man*. *bracodemoca* Woodland, 1935 (type-species) and *Man*. *piscatorum* (de Chambrier & Vaucher, 1999) (= *Mar*. *piscatorum*), while *Ageneiella* was synonymized with *Mariauxiella*, which currently includes two species, i.e., *Mar*. *pimelodi* de Chambrier & Rego, 1995 (type-species) and *Mar*. *brevifilis* (de Chambrier & Vaucher, 1999) (= *Ageneiella brevifilis*).

As part of ongoing efforts to improve our understanding of the diversity, phylogenetic interrelationships and host-parasite associations of proteocephalids, tapeworms collected from *Ageneiosus militaris* Valenciennes (Siluriformes: Auchenipteridae) from the Upper Paraná River basin, State of São Paulo, Brazil, were subjected to morphological, ultrastructural, and molecular phylogenetic analyses. The evidence gathered indicates that these tapeworms represent a new species of *Mariauxiella*, which is described herein.

## Materials and methods

### Sample collection

Twenty-six specimens of *A*. *militaris* were collected in the lowermost Pardo River (22°54′41.6″S 49°57′39.9″W), near its discharge into the Paranapanema River, in the municipality of Salto Grande, State of São Paulo, Brazil. Hosts were euthanized by pithing and had their intestines screened for cestodes.

The tapeworms were removed from the host’s intestine, placed in saline, and gently cleaned of intestinal contents. The posteriormost proglottides were excised and fixed in 96% molecular-grade ethanol for molecular analyses. The remaining strobila with the scolex (hologenophores *sensu* Pleijel et al., 2008) were placed in a small amount of saline and heat-killed with almost boiling 4% formaldehyde solution. After two weeks, formalin-fixed specimens were transferred to 70% ethanol before morphological and ultrastructural analyses.

### Morphological and ultrastructural data

Tapeworms were processed following the guidelines of Chervy (2024). The specimens were stained with Mayer’s hydrochloric carmine solution, dehydrated in an ethanol series, cleared with eugenol (clove oil) and mounted in Canada balsam. For histological sections, a few proglottides were embedded in paraffin wax, cross-sectioned, and stained with haematoxylin-eosin. For scanning electron microscopy (SEM) analyses, the scolex and two proglottides of the holotype were dehydrated through a graded ethanol series, dried in hexamethyldisilazane, coated with gold and examined in a FEI Quanta 200 scanning-electron microscope at the Centro de Microscopia Eletrônica, Instituto de Biociências, Universidade Estadual Paulista ‘Júlio de Mesquita Filho’ (UNESP), Botucatu.

Photomicrographs and measurements were taken using the Leica Application Suite software LAS V3 connected to a Leica DMLB 5000 (Leica Microsystems, Wetzlar, Germany). Detailed line drawings were made using a Leica DM 2500 LED with an attached drawing tube and digitized using the tablet-based drawing software Procreate v.5.4.10.

Microthrix terminology follows Chervy (2009) and the fish names follow Froese & Pauly (2026). All measurements in descriptions are given in micrometres unless otherwise indicated. Abbreviations used are: x – mean; n – number of measurements; SG – host field code (= Salto Grande, Brazil); CHIOC – Helminthological Collection of the Instituto Oswaldo Cruz, Rio de Janeiro, Brazil.

### Molecular data

Posteriormost proglottides of two specimens were used for the genomic DNA extraction. PCR amplification of the large nuclear ribosomal RNA subunit (RNA28S; D1–D3 domains), and the complete mitochondrial cytochrome *c* oxidase subunit I (MT-CO1) were performed following Alves et al. (2021a). PCR products were purified using magnetic beads Agencourt AMPure XP (Beckman Coulter, Inc.), and Sanger sequenced on an Applied Biosystems ABI 3500 DNA genetic analyzer. Contiguous sequences were assembled and inspected for errors using Geneious Prime v.2024.0.5, and their consensus were submitted to GenBank.

The newly generated sequences were assembled into two alignments (RNA28S and MT-CO1 markers) together with selected members of the Clade VIII of Alves et al. (2025), mostly composed of fish proteocephalids from the Neotropical region (92 publicly available sequences; Table 1). The alignments were performed using ‘--auto’ command of MAFFT v.7.490 (Katoh et al., 2002; Katoh & Standley, 2013) implemented in Geneious; unreliable positions were identified and removed using the Gblock web server (https://ngphylogeny.fr/; Dereeper et al., 2008) with less stringent settings. Final alignments of the RNA28S (1,423 bp long; Online Resource 1) and MT-CO1 (1,608 bp long; Online Resource 2) genes were concatenated in a larger matrix 3,031 bp long (Online Resource 3). Outgroup was chosen based on a wider phylogenetic analysis of Alves et al. (2025).

**Table 1:** GenBank accession numbers of DNA sequences of proteocephalids (Proteocephalidae) used in the phylogenetic analyses. New sequences are in bold.

Taxon	Host species (family)	Accession number		References
		RNA 28S	MT-CO1	
**INGROUP**				
*Amphoteromorphus ninoi* Carfora, de Chambrier & Vaucher, 2003	*Brachyplatystoma vaillantii* (Pimelodidae)	PX283256	PX395807	Alves et al. (2026)
*Amphoteromorphus ovalis* Carfora, de Chambrier & Vaucher, 2003	*Brachyplatystoma filamentosum* (Pimelodidae)	PX283237	PX326274	Alves et al. (2026)
*Amphoteromorphus parkamoo* Woodland, 1935	*Zungaro zungaro* (Pimelodidae)	PX283280	PX326298	Alves et al. (2026)
*Amphoteromorphus piraeeba* Woodland, 1934	*Brachyplatystoma filamentosum* (Pimelodidae)	PX283236	PX395801	Alves et al. (2026)
*Brayela karuatayi* (Woodland, 1934)	*Platynematichthys notatus* (Pimelodidae)	PX283283	PX326300	Alves et al. (2026)
*Brooksiella praeputialis* (Rego, Santos & Silva, 1974)	*Cetopsis coecutiens* (Cetopsidae)	AJ275229	PQ340940	de Chambrier et al. (2004); Alves et al. (2025)
*Chambriella megacephala* (Woodland, 1934)	*Sorubimichthys planiceps* (Pimelodidae)	KY207449	PQ350129	Alves et al. (2017b); Alves et al. (2025)
*Choanoscolex abscisus* (Riggenbach, 1895)	*Pseudoplatystoma corruscans* (Pimelodidae)	PQ343985	PQ340965	Alves et al. (2025)
*Cordicestus rafaeli* Scholz, de Chambrier & Kyslík, 2024	*Atractosteus tropicus* (Lepisosteidae)	PP768088	PP803427	Scholz et al. (2024)
*Cordicestus rafaeli* Scholz, de Chambrier & Kyslík, 2024	*Atractosteus tropicus* (Lepisosteidae)	PX283229	PX326272	Alves et al. (2026)
*Cordicestus singularis* (La Rue, 1911)	*Lepisosteus oculatus* (Lepisosteidae)	PP768089, PP768090	PP803375, PP782045	Scholz et al. (2024)
*Cordicestus* sp. 2	*Lepisosteus oculatus* (Lepisosteidae)	PP768086	PP782046	Scholz et al. (2024)
*Endorchis piraeeba* Woodland, 1934	*Brachyplatystoma filamentosum* (Pimelodidae)	PX283282	PX326299	Alves et al. (2026)
*Gibsoniela meursaulti* de Chambrier & Vaucher, 1999	*Ageneiosus inermis* (Auchenipteridae)	MW798190, PX283257	MW811139, PX326316	Alves et al. (2021b); Alves et al. (2026)
*Goezeella siluri* Fuhrmann, 1916	*Pinirampus pirinampu* (Pimelodidae)	MF370207	PQ340963	Alves et al. (2017c); Alves et al. (2025)
*Harriscolex nathaliae* Gil de Pertierra & de Chambrier, 2013	*Pseudoplatystoma corruscans* (Pimelodidae)	PX283248, PQ343984	PX326282, PQ340962	Alves et al. (2025); Alves et al. (2026)
*Houssayela sudobim* (Woodland, 1935)	*Pseudoplatystoma punctifer* (Pimelodidae)	PX283238	PX326275	Alves et al. (2026)
*Jauella glandicephala* Rego & Pavanelli, 1985	*Zungaro zungaro* (Pimelodidae)	PX283253	PX326286	Alves et al. (2026)
*Laruella perplexa* (La Rue, 1911)	*Amia calva* (Amiidae)	PP768082, PP768083	PP782037, PP782038	Scholz et al. (2024)
*Manaosia bracodemoca* Woodland, 1935	*Sorubim lima* (Pimelodidae)	MW798193, MW798194	MW811143, MW811144	Alves et al. (2021b)
*Manaosia piscatorum* (de Chambrier & Vaucher, 1999)	*Hemisorubim platyrhynchos* (Pimelodidae)	MW798195, MW798196	MW811141, MW811142	Alves et al. (2021b)
*Mariauxiella brevifilis *(de Chambrier & Vaucher, 1999)	*Ageneiosus inermis* (Auchenipteridae)	MW798189, PX283251, PX283279	MW811145, PX326285, PX395815	Alves et al. (2021b); Alves et al. (2026)
*Mariauxiella pimelodi* de Chambrier & Rego, 1995	*Pimelodus ornatus* (Pimelodidae)	MW244589, PX283271	MW244031, PX326295	Alves et al. (2021a); Alves et al. (2026)
***Mariauxiella saltogrande* n. sp.**	***Ageneiosus militaris* (Auchenipteridae)**	**PZ704327, PZ704328**	**PZ704266, PZ704267**	**Present study**
*Monticellia magna* (Rego, Santos & Silva, 1974)	*Pimelodus maculatus* (Pimelodidae)	PQ343981, PQ343982	PQ340959, PQ340960	Alves et al. (2025)
*Monticellia ophisterni* Scholz, de Chambrier & Salgado-Maldonado, 2001	*Ophisternon aenigmaticum* (Synbranchidae)	PX283244	PX326279	Alves et al. (2026)
*Monticellia ventrei* de Chambrier & Vaucher, 1999	*Pinirampus pirinampu* (Pimelodidae)	PQ343980, PX283275	PQ340958, PX395812	Alves et al. (2025); Alves et al. (2026)
*Nomimoscolex admonticellia* (Woodland, 1934)	*Pinirampus pirinampu* (Pimelodidae)	PQ343979, PX283270	PQ340957, PX326294	Alves et al. (2025); Alves et al. (2026)
*Nomimoscolex lopesi* Rego, 1989	*Pseudoplatystoma punctifer* (Pimelodidae)	PX283262	PX326288	Alves et al. (2026)
*Nomimoscolex microacetabula* Gil de Pertierra, 1995	*Pimelodus maculatus* (Pimelodidae)	MW244595, MW244597	MW244037, MW244038	Alves et al. (2021a)
*Nomimoscolex sudobim* Woodland, 1935	*Pseudoplatystoma* cf. *reticulatum* (Pimelodidae)	PQ343978	PQ340956	Alves et al. (2025)
*Ophiotaenia currani* de Chambrier, Kudlai, McAllister & Scholz, 2023	*Nerodia fasciata confluens* (Colubridae)	OR663966	OR660032	de Chambrier et al. (2023)
*Ophiotaenia europaea* Odening, 1963	*Natrix tessellata* (Colubridae)	PX283221	PX326269	Alves et al. (2026)
*Ophiotaenia grandis* La Rue, 1911	*Agkistrodon piscivorus* (Viperidae)	OR663968	OR671430	de Chambrier et al. (2023)
*Ophiotaenia perspicua* La Rue, 1911	*Nerodia rhombifer* (Colubridae)	OR663967	OR671429	de Chambrier et al. (2023)
*Ophiotaenia sanbernardinensis* Rudin, 1917	*Helicops leopardinus* (Colubridae)	PX283252	PX395805	Alves et al. (2026)
*Ophiotaenia saphena* Osler, 1931	*Lithobates clamitans* (Ranidae)	OQ168417, OQ168418	OQ200060, OQ200061	Scholz et al. (2023)
*Ophiotaenia tkachi* de Chambrier, Kudlai, McAllister & Scholz, 2023	*Nerodia fasciata confluens* (Colubridae)	OR663962	OR660028	de Chambrier et al. (2023)
*Ophiotaenia tkachi* de Chambrier, Kudlai, McAllister & Scholz, 2023	*Agkistrodon piscivorus* (Viperidae)	OR663963	OR660029	de Chambrier et al. (2023)
*Ophitaenia* sp.	*Lithobates pipiens* (Ranidae)	OQ168422, OQ168423	OQ200065, OQ200066	Scholz et al. (2023)
*Peltidocotyle* sp.	*Zungaro zungaro* (Pimelodidae)	PX283265	PX326290	Alves et al. (2026)
*Peltidocotyle* sp.	*Brachyplatystoma vaillantii* (Pimelodidae)	PX283259	PX395808	Alves et al. (2026)
*Proteocephalus ambloplitis* (Leidy, 1887)	*Micropterus dolomieu* (Centrarchidae)	PP768095	PP803376	Scholz et al. (2024)
*Proteocephalus bagri* Holcman-Spector & Mañé-Garzón, 1988	*Rhamdia quelen* (Heptapteridae)	PX283246	PX326281	Alves et al. (2026)
*Proteocephalus pimelodi* (Gil de Pertierra, 1995)	*Pimelodus albicans* (Pimelodidae)	PX283249	PX326283	Alves et al. (2026)
*Proteocephalus regoi* de Chambrier, Scholz & Vaucher, 1996	*Hoplias malabaricus* (Erythrinidae)	PX283250	PX326284	Alves et al. (2026)
*Proteocephalus rhamdiae* Holcman-Spector & Mañé-Garzón, 1988	*Rhamdia quelen* (Heptapteridae)	PX283234	PX326273	Alves et al. (2026)
*Proteocephalus sophiae* de Chambrier & Rego, 1994	*Zungaro zungaro* (Pimelodidae)	PX283269	PX326293	Alves et al. (2026)
*Pseudoendorchis auchenipteri* (de Chambrier & Vaucher, 1999)	*Pimelodus maculatus* (Pimelodidae)	MW244599	MW244040	Alves et al. (2021a)
*Pseudoendorchis auchenipteri* (de Chambrier & Vaucher, 1999)	*Auchenipterus osteomystax* (Auchenipteridae)	MW244603	MW244044	Alves et al. (2021a)
*Pseudoendorchis cristata* Alves, de Chambrier, Luque & Scholz, 2020	*Pimelodella cristata* (Heptapteridae)	MW244604, MW244605	MW244045, MW244046	Alves et al. (2021a)
*Pseudoendorchis felipei* Alves, de Chambrier, Luque & Scholz, 2020	*Pimelodus fur* (Pimelodidae)	MW244607, MW244608	MW244048, MW244049	Alves et al. (2021a)
*Pseudoendorchis megalonemi* Alves, de Chambrier & Scholz, 2021	*Megalonema platycephalum* (Pimelodidae)	MW244616	MW244057	Alves et al. (2021a)
*Pseudoendorchis megalonemi* Alves, de Chambrier & Scholz, 2021	*Megalonema platycephalum* (Pimelodidae)	PX283284	PX326301	Alves et al. (2026)
*Pseudoendorchis souzalimae* Alves, de Chambrier, Luque & Scholz, 2020	*Pimelodus maculatus* (Pimelodidae)	MW244609, MW244610	MW244050, MW244051	Alves et al. (2021a)
*Pseudoendorchis* sp. 1	*Pimelodella* sp. (Auchenipteridae)	MW244611	MW244052	Alves et al. (2021a)
*Pseudoendorchis* sp. 1	*Pimelodus maculatus* (Pimelodidae)	MW244612	MW244053	Alves et al. (2021a)
*Pseudoendorchis* sp. 2	*Pimelodus maculatus* (Pimelodidae)	MW244613, MW244614	MW244054, MW244055	Alves et al. (2021a)
*Pseudoendorchis* sp. 3	*Pimelodus maculatus* (Pimelodidae)	PQ343969	PQ340947	Alves et al. (2025)
*Riggenbachiella amazonense* Alves, de Chambrier, Luque & Scholz, 2017	*Sorubimichthys planiceps* (Pimelodidae)	MN626407	MN624071	Alves et al. (2019)
*Riggenbachiella paranaense* (Pavanelli & Rego, 1989)	*Hemisorubim platyrhynchos* (Pimelodidae)	MN626405, MN626406	MN624072, MN624073	Alves et al. (2019)
*Spasskyellina lenha* (Woodland, 1933)	*Sorubimichthys planiceps* (Pimelodidae)	PX283286, PX283291	PX326302, PX326306	Alves et al. (2026)
*Spasskyellina mandi* Pavanelli & Takemoto, 1996	*Pimelodus ornatus* (Pimelodidae)	MW244618, MW244619	MW244058, MW244059	Alves et al. (2021a)
*Spasskyellina spinulifera* (Woodland, 1935)	*Pseudoplatystoma reticulatum* (Pimelodidae)	PQ343965, PQ343966	PQ340943, PQ340944	Alves et al. (2025)
*Spatulifer maringaensis* Pavanelli & Rego, 1989	*Sorubim lima* (Pimelodidae)	PX283287, PX283292	PX326303, PX326307	Alves et al. (2026)
*Synbranchiella mabelae* Arredondo, Alves & Gil de Pertierra, 2017	*Synbranchus marmoratus* (Synbranchidae)	KY798870	PQ340941	Arredondo et al. (2017); Alves et al. (2025)
*Travassiella jandia* (Woodland, 1934)	*Zungaro zungaro* (Pimelodidae)	PX283242	PX326277	Alves et al. (2026)
**OUTGROUP**				
*Proteocephalus macrophallus* (Diesing, 1850)	*Cichla monoculus* (Cichlidae)	PX283254	PX326287	Alves et al. (2026)
*Proteocephalus microscopicus* Woodland,1935	*Cichla monoculus* (Cichlidae)	PX283277	PX395813	Alves et al. (2026)

Phylogenetic reconstructions were performed with the maximum likelihood (ML) criterion for all datasets (RNA28S, MT-CO1, and combined RNA28S + MT-CO1), whereas Bayesian inference (BI) was applied only to the concatenated one. The best evolutionary models were estimated using ModelFinder (Kalyaanamoorthy et al., 2017) within IQ-TREE v.2.3.6 (Minh et al., 2020), based on the small sample size corrected Akaike information criterion (AICc). The model GTR+F+I+G4 was chosen for the individual and concatenated datasets with two partitions.

The ML trees were generated using IQ-TREE and branch supports were estimated with 1,000 replicates of the ultrafast bootstrap (UFBoot) (Hoang et al., 2018) and 1,000 SH-aLRT tests (Guindon et al., 2010). To avoid overestimation of UFBoot, we used a hill-climbing nearest-neighbor interchange (NNI) (Hoang et al., 2018). BI analyses were performed in MrBayes v.3.2.7a (Ronquist et al., 2012), running two independent MC3 runs of four chains for 10 million generations, sampling tree topologies every 1000th generation, with the first 25% of samples discarded as burn-in. We used Tracer v.1.7.2 (Rambaut et al., 2018) to check the convergence and mixing of parameters and to confirm that individual effective sample size (ESS) was adequate to provide reasonable estimates of the variance in model parameters. Nucleotide divergences among taxa were calculated as uncorrected *p*-distances in Geneious.

**Proteocephalidae La Rue, 1911**

***Mariauxiella*** de Chambrier & Rego, 1995

Type species: *Mariauxiella pimelodi* de Chambrier & Rego, 1995, by original designation

***Mariauxiella saltogrande*** **n. sp.**

*Type-host*: *Ageneiosus militaris* Valenciennes (Siluriformes: Auchenipteridae).

*Type-locality*: Lowermost Pardo River (22°54′41.6″S 49°57′39.9″W; tributary of the Paranapanema River, Upper Paraná River basin), municipality of Salto Grande, State of São Paulo, Brazil.

*Type material*: Holotype – specimen on one slide (scolex and two proglottides observed using SEM) and cross sections on two slides [SG-34A; CHIOC 41117a–c (hologenophore)]; paratypes – two complete specimens [SG24A, SG 34B; CHIOC 41119 (hologenophore), and CHIOC 41118, respectively]. All specimens were collected by P. V. Alves and R. J. da Silva on 11 and 12 October 2022.

*Other material*: Two immature, incomplete specimens (SG23A, B; not deposited).

*Site of infection*: Anterior intestine.

*Prevalence*: 12% (3/26). Two host specimens harbored two tapeworms each, whereas a single cestode was found in the remaining infected host.

*Representative DNA sequences*: Two identical partial RNA 28S sequences (length of 1,399 bp; PZ704327, PZ704327) and two identical complete MT-CO1 sequences (length of 1,617 bp, encoding 538 amino acids; PZ704266, PZ704267).

*ZooBank registration*: The Life Science Identifier (LSID) for *Mariauxiella saltogrande* **n. sp.** is urn:lsid:zoobank.org:act:9C96E959-80D4-4AF6-AA74-35658E12B3AD. The publication is also registered in ZooBank: urn:lsid:zoobank.org:pub:DDB2C589-3315-4B01-BD0E-1FDBB074B749

*Etymology*: The specific name refers to the municipality of Salto Grande, State of São Paulo, Brazil, through which the Pardo River flows and where the fish hosts were collected. The name is treated as a noun in apposition.

### Description (Figs. 1–3)

**Fig. 1:**
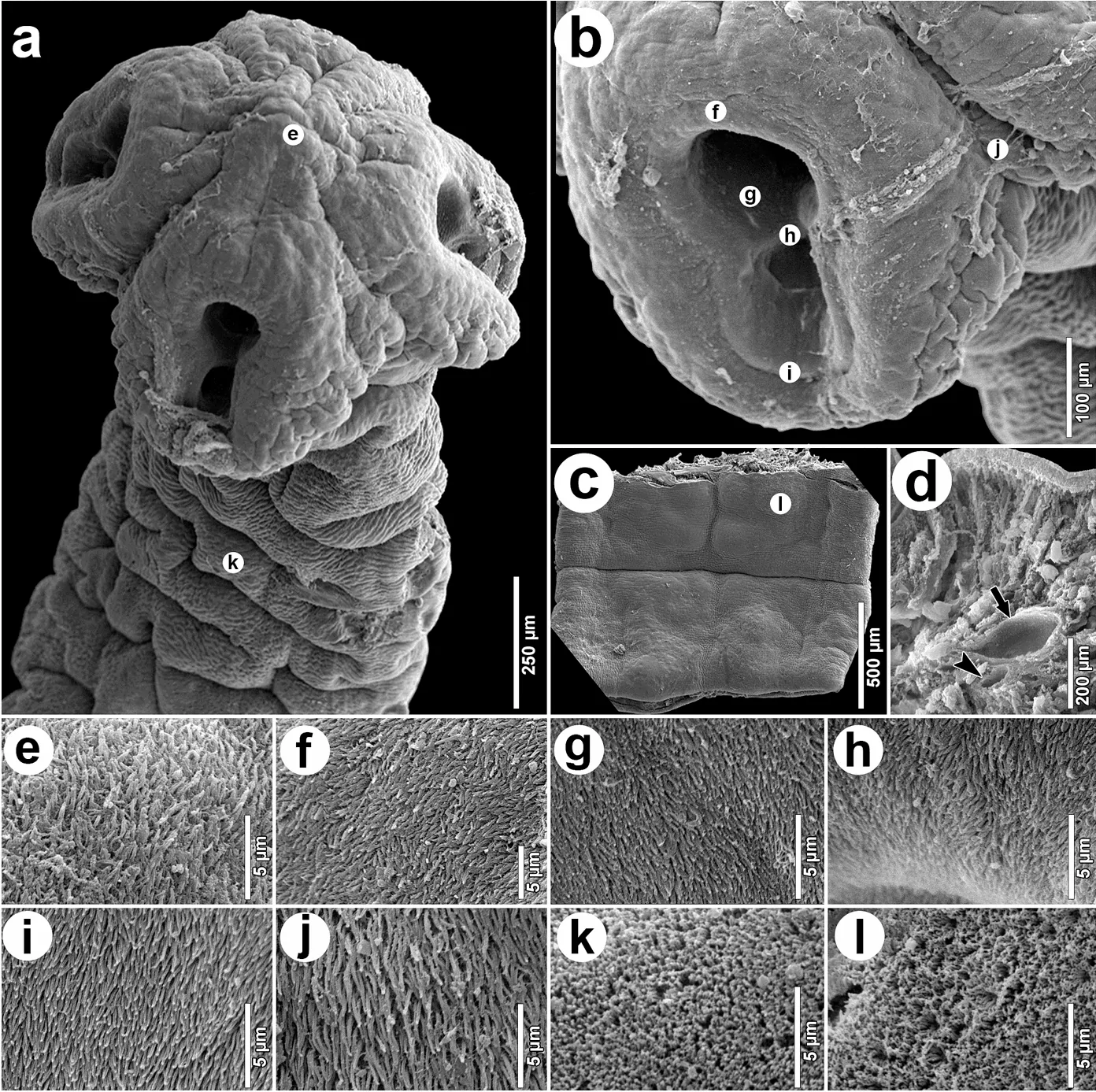
Scanning electron micrographs of *Mariauxiella saltogrande* **n. sp.** from *Ageneiosus militaris*, Brazil (holotype, CHIOC 41117). (**a**) Scolex and proliferative zone, dorsolateral view (note the longitudinal and transverse grooves on the tegument). (**b**) Detail of biloculate suckers and free posterior margin. (**c**) mature proglottides, dorsoventral view. (**d**) Sectioned proglottis showing the ventral (arrow) and dorsal (arrowhead) osmoregulatory canals. (**e**–**l**) Microtriches on the apex of scolex, upper rim, luminal surface, septum and lower rim of suckers, surface between suckers, proliferative zone, and proglottis, respectively.

**Fig. 2:**
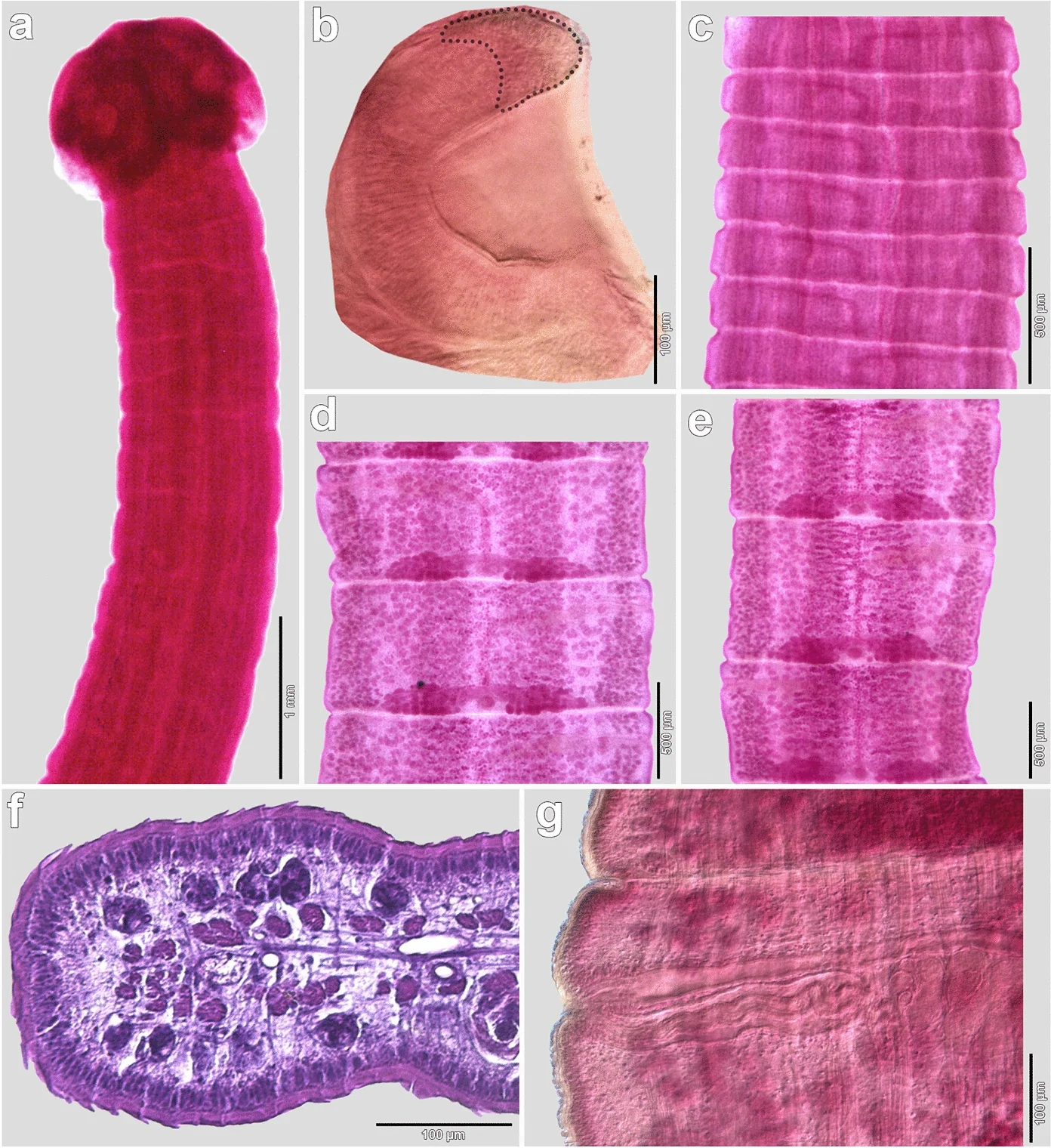
Light photomicrographs of *Mariauxiella saltogrande* **n. sp.** from *Ageneiosus militaris*, Brazil. (**a**) Scolex and proliferative zone, dorsoventral view (paratype, CHIOC 41119). (**b**) Detail of anterior sucker loculus showing the distal, well-developed circular musculature (dotted area) (paratype, CHIOC 41118). (**c–e**) Immature, pregravid and gravid proglottides, dorsal view (holotype, CHIOC 41117). (**f**) Cross-section at aporal region of a proglottis (holotype). (**g**) Terminal genitalia, dorsal view (holotype).

**Fig. 3:**
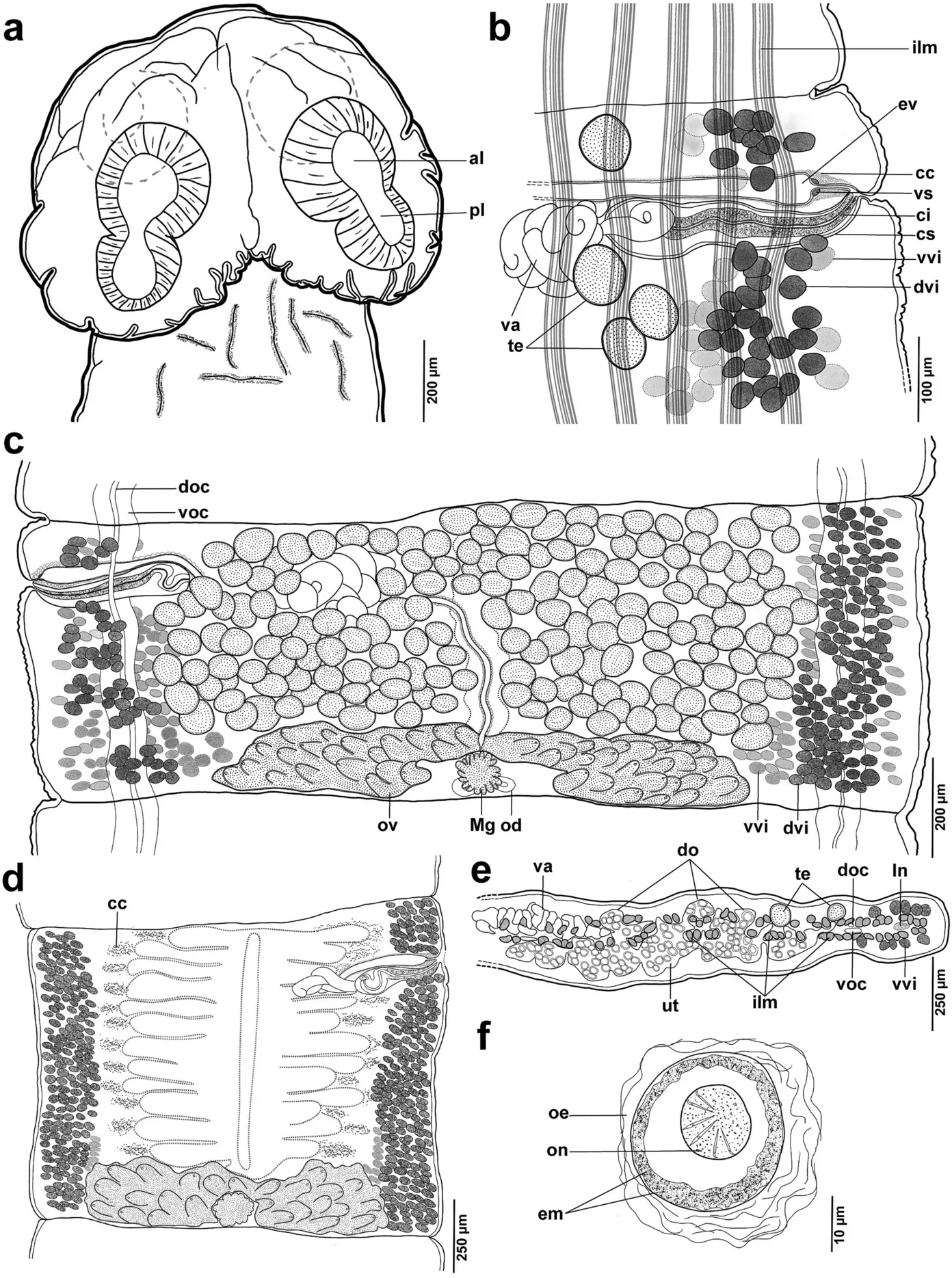
Line drawings of *Mariauxiella saltogrande* **n. sp.** from *Ageneiosus militaris*, Brazil. (**a**) Scolex, dorsoventral view (paratype, CHIOC 41119). (**b**) Terminal genitalia, dorsal view (holotype, CHIOC 41117). (**c**) Mature proglottis, dorsal view (holotype). (**d**) Gravid proglottis, dorsal view (vas deferens and vaginal canal intentionally omitted). (**e**) Cross-section of gravid proglottis at cirrus-sac level (holotype). (**f**) Egg (holotype). *Abbreviations*: al, anterior loculus; cc, chromophilic cells; ci, cirrus; cs, cirrus-sac; doc, dorsal osmoregulatory canal; do, dorsal outgrowths; dvi, dorsal vitelline follicles; em, embryophore; ev, enlarged vaginal canal; ilm, inner longitudinal musculature; ln, longitudinal nerve; Mg, Mehlis’ gland; od, oviduct; oe, outer envelope; on, oncosphere; ov, ovary; pl, posterior loculus; te, testes; ut, uterus; va, vas deferens; voc, ventral osmoregulatory canal; vs, vaginal sphincter; vvi, ventral vitelline follicles.

(Based on three specimens). Medium-sized worm, 80–92 mm long, up to 1.78 mm wide. Tegument with longitudinal and transverse grooves, more conspicuous on scolex and proliferative zone (neck) (Figs. 1a, c, 2a). Strobila acraspedote, anapolytic (Figs. 1c, 2c–e, 3c, d), consisting of about 180–240 proglottides: 110–135 immature, 15–25 mature, and 55–80 pregravid and gravid proglottides. Immature and mature proglottides much wider than long (length: width ratio 0.15–0.54), pregravid and gravid proglottides wider than long (length: width ratio 0.46–0.78).

Scolex massive, quadrilobed, 640–835 long and 930–1,180 wide, much wider than proliferative zone 725–920 wide, bearing four biloculate suckers directed antero-laterally (Figs. 1a, b, 2a, 3a); apex slightly conical, apical organ and metascolex absent (Figs. 1a, 2a, 3a). Suckers conspicuous, with free posterior margin; anterior loculus 245–300 × 275–320 larger than posterior loculus 160–205 × 185–260 (Figs. 1a, b, 2a, 3a), with well-developed circular musculature at upper and lower marginal regions (Fig. 2b). Apex of scolex, upper and lower rim, septum, and luminal surface of suckers covered with acicular filitriches, interspersed with gladiate spinitriches becoming more numerous towards lower rim of suckers (Fig. 1e–j). Proliferative zone and strobila covered with acicular filitriches of similar appearance and density (Fig. 1k, l).

Inner longitudinal musculature well developed, formed by numerous large bundles of muscle fibres, clearly visible in whole-mounts (Figs. 2f, g, 3b, e). Two pairs of osmoregulatory canals slightly sinuous, situated at same level, overlapping cirrus-sac and rows of vitelline follicles, lateral to lateralmost testes (Figs. 1d, 2f, 3c, e).

Testes cortical, spherical, 40–60 in diameter, arranged in up to three layers, forming one dorsal field between anterior margin of proglottides and anterior ovarian lobes, 188–256 in number (x = 221, n = 5), absent or less numerous at level of lower and middle portions of uterine stem in mature and pregravid proglottides; testes partially overlap vitelline follicles, rarely reaching osmoregulatory canals (Figs. 2d, e, g, 3b, c, e). Vas deferens strongly coiled, never crossing median line of proglottides (Figs. 2d, e, 3b, c, e). Cirrus-sac elongated, 235–350 (x = 298) long and 50–75 (x = 58) wide (n = 13), its length representing 17–23% (x = 19%, n = 13) of proglottis width; cirrus almost straight, reaching up to 59% of cirrus-sac length (Figs. 2g, 3b, c). Genital pores alternating irregularly, markedly pre-equatorial, situated at 15–23% (x = 18%, n = 13) of proglottis length from anterior margin (Figs. 2d, e, g, 3b–d); common genital atrium narrow, shallow (Fig. 3b–d).

Ovary bilobed, occupying 59–70% (x = 64%, n = 13) of proglottis width; its length represents 18–24% (x = 21%, n = 13) of proglottis length (Figs. 2d, e, 3c, d). Ovarian lobes penetrating to dorsal cortex not seen. Relative ovarian size, i.e., ratio of surface of ovary to surface of proglottis (see de Chambrier et al., 2012), 7–13% (x = 11%, n = 12). Mehlis’ gland 65–145 long and 75–180 wide, representing 4–8% of proglottis width (x = 6%, n = 12) (Figs. 2d, e, 3c, d). Vaginal canal slightly sinuous, with symmetrically enlarged (chamber-like) terminal part (*pars copulatrix vaginae*), abruptly narrowing at level of small vaginal sphincter surrounded by conspicuous concentration of chromophilic cells; vagina always anterior to cirrus-sac (Figs. 2g, 3b–d). Vitelline follicles cortical, arranged in crescent lateral rows on each side, occupying almost entire proglottis length; more numerous and widely distributed ventrally, towards ovary (Figs. 2d, e, 3b–e).

Uterus cortical, with 12–18 lateral diverticula on each side bearing several outgrowths crossing entire medulla to dorsal cortex (Fig. 3e); development of type 3 according to Alves et al. (2021b) (see also de Chambrier & Rego, 1995). Eggs spherical (Fig. 3f), with bilayered embryophore 26–29 in diameter; oncosphere spherical, 13–16 in diameter; hooks 6–8 long.

### Remarks

The new species was assigned to *Mariauxiella* based on the following combination of characters: a massive scolex with biloculate suckers bearing strong circular musculature, well-developed internal longitudinal musculature, vagina always anterior to the cirrus-sac with a symmetrical, enlarged terminal part abruptly narrowing at the level of the vaginal sphincter, vitelline follicles arranged in crescent-shaped lateral rows on each side, and lateral uterine diverticula projecting multiple outgrowths that cross the entire medulla to the dorsal cortex (uterine development type 3) (Alves et al., 2021b).

The most distinctive feature distinguishing the two previously known species of *Mariauxiella* is the number of loculi in the suckers. Whereas *Mar*. *pimelodi* possesses uniloculate suckers with double crescent-shaped circular musculature, *Mar*. *brevifilis* exhibits biloculate suckers with circular musculature restricted to the upper and lower marginal portions (Table 2; de Chambrier & Rego, 1995; de Chambrier & Vaucher, 1999). *Mariauxiella saltogrande* **n. sp.** resembles *Mar*. *brevifilis* by also having biloculate suckers and a similar arrangement of the circular musculature. However, the species differ in several features, including body size (length 80–92 mm *vs* 22–49 mm, and maximum width 1.78 mm *vs* 5.1 mm), scolex shape (quadrilobed *vs* rectangular in *Mar*. *brevifilis*; compare fig. 98 of de Chambrier & Vaucher, 1999 and Fig. 1a), and arrangement of the osmoregulatory canals (ventral and dorsal canals overlapping each other *vs* ventral canal median to dorsal canal; compare fig. 29 of de Chambrier & Vaucher, 1999 and Figs. 1d, 3c) (see Table 2). New molecular data also support *Mariauxiella saltogrande* **n. sp.** as an independent lineage of the monophyletic *Mariauxiella* (see below).

**Table 2:** Selected morphological and morphometric traits of *Mariauxiella* spp. from Neotropical catfishes (measurements in micrometers unless otherwise stated).

Cestode species	*Mariauxiella pimelodi*	*Mariauxiella brevifilis*	*Mariauxiella saltogrande* **n. sp.**
Fish host (family)	*Pimelodus* sp., *P*. *ornatus* (Pimelodidae)	*Ageneiosus inermis* (Auchenipteridae)	*Ageneiosus militaris* (Auchenipteridae)
Locality (country)	Cuiabá and Paraná Rivers (Brazil); Pirapo and Paraguay Rivers (Paraguay)	Jejuí Guazú and Paraguay Rivers (Paraguay)	Lowermost Pardo River (Brazil)
Reference	de Chambrier and Rego (1995)	de Chambrier and Vaucher (1999)	This study
Sample size	n = 6	n = 13	n = 3
Body length (mm)	30–50	22–49	80–92
Body width (mm)	up to 1.05	up to 5.1	up to 1.78
Proglottis shape (pregravid)	longer than wide	wider than long	wider than long
Scolex shape	massive, rectangular	massive, rectangular	massive, quadrilobed
Scolex length	1,110–1,520 diameter	1,355–1,915 diameter	640–835
Scolex width	–	–	930–1,180
Number SL	one loculus	two loculi	two loculi
Shape of CM in suckers	hypertrophied, double crescent (banana-shaped)	well-developed on the upper and lower marginal region	well-developed on the upper and lower marginal region
Comparative loculi size	–	anterior larger than posterior	anterior larger than posterior
CSL/PW cirrus-sac level (%)	19–25	10–19	17–23
OW/PW ovary level (%)	65–77	58–69	59–70
Testes number	105–172	244–286	188–256
OC arrangement	overlapping each other	ventral OC median to dorsal one	overlapping each other
GPD/PL (%)	11–18	15–25	15–23
VL/PL both sides (%)	*c*. 100%	*c*. 100%	*c*. 100%

*Abbreviations*: CM, circular musculature; SL, sucker loculi; OC, osmoregulatory canals; CSL/PW, cirrus-sac length/proglottis width; OW/PW, ovarian width/proglottis width; GPD/PL, distance of genital pore to anterior margin of proglottis/proglottis length; VL/PL, length of lateral rows of vitelline follicles/proglottis length.

## Molecular analyses

**Fig. 4:**
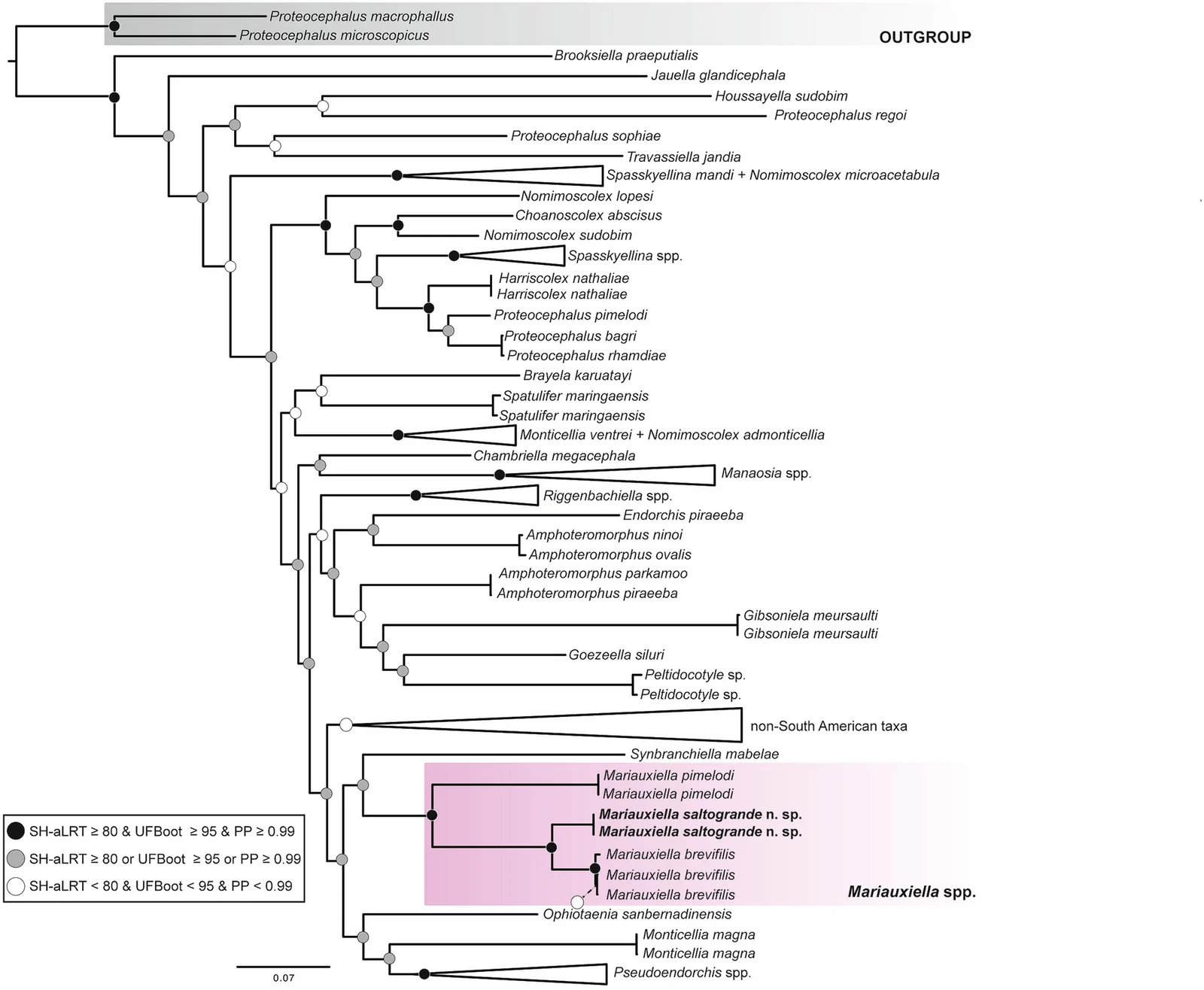
Maximum likelihood phylogram based on combined RNA 28S + MT-CO1 datasets (3,031 bp long) of selected Proteocephalidae including highlighted clade of *Mariauxiella* spp. Shimodaira-Hasegawa approximate likelihood ratio test (SH-aLRT), ultrafast bootstrap (UFBoot), and posterior probability (PP) branch support values are shown at nodes (see inset). Newly generated sequences are in bold. Branch length scale bar indicates number of substitutions per site.

Phylogenetic analyses based on individual [Online Resources 4 (RNA28S) and 5 (MT-CO1)] and combined RNA28S + MT-CO1 datasets (Fig. 4; Online Resource 6) revealed a sister relationship between *Mariauxiella saltogrande* **n. sp.** and *Mar. brevifilis*. In turn, both species formed a clade together with the type species and the only other species in the genus, *Mar. pimelodi*. The close relationship between the new species and *Mar*. *brevifilis*, as well as the monophyly of *Mariauxiella*, were strongly supported in all analyses, whereas their interrelationships with other Neotropical proteocephalids remained uncertain (Fig. 4). Pairwise comparison of the RNA28S and MT-CO1 sequence data revealed interspecific divergence among *Mariauxiella* species of 0.18–1.07% (3–15 nt) and 7.8–15.1% (124–243 nt), respectively, whereas only *Mar*. *brevifilis* showed some degree of intraspecific variation, ranging from 0.11–0.18 (1–3 nt) for RNA28S and 0.06–0.81 (1–13 nt) for MT-CO1.

## Discussion

Using an integrative taxonomic framework, the present study recognizes *Mariauxiella saltogrande* **n. sp.** as a new species based on newly collected material from the auchenipterid catfish *Ageneiosus militaris* in the Upper Paraná River basin in Brazil. Although only a few specimens were available, they provided compelling morphological, ultrastructural, and molecular evidence supporting the establishment of this new taxon, expanding our current knowledge of species diversity within *Mariauxiella*. With the addition of *Mariauxiella saltogrande* **n. sp.**, the genus now comprises three species parasitizing pimelodid and auchenipterid catfishes in South America.

The genus *Mariauxiella* has recently been revised to better reflect the evolutionary history of its species (Alves et al., 2021b). This reappraisal demonstrated that the similarities between *Mariauxiella pimelodi* and *Manaosia piscatorum* (previously *Mariauxiella piscatorum*) evolved independently, whereas those shared by *Mar*. *pimelodi* and *Mar*. *brevifilis* were inherited from a common ancestor (see Alves et al., 2021b for details). Likewise, the morphological similarities shared between these two species and *Mariauxiella saltogrande* **n. sp.** likely represent homologous traits, as supported by their close phylogenetic relationship (see Fig. 4). The differentiation between *Mar*. *pimelodi* and the two other congeners is straightforward, as the type species has a scolex bearing uniloculate suckers, while *Mar*. *brevifilis* and the new species have biloculate suckers. Congeneric species having different numbers of sucker loculi are rare among proteocephalids and, in fact, were only recognized after molecular evidence became available (Alves et al., 2021a, b). Furthermore, *Mar*. *pimelodi* is the only species known from pimelodid catfishes (*Pimelodus ornatus* Kner), whereas *Mar*. *brevifilis* and *Mariauxiella saltogrande* **n. sp.** parasitize *Ageneiosus inermis* Linnaeus and *A*. *militaris*, respectively, both auchenipterid catfishes. *Mariauxiella saltogrande* **n. sp.** differs from *Mar. brevifilis*, and is mainly characterized, by having a longer but narrower body and a scolex with four well-delimited suckers that give it a distinctly quadrilobate appearance, rather than the deeply embedded suckers lacking a free posterior margin that result in a rectangular appearance observed in *Mar. brevifilis.* Differences in the arrangement of the osmoregulatory canals between the two species (see Remarks) also affect the distribution of the testes, which rarely reach the osmoregulatory canals in the new species but extend beyond them in *Mar*. *brevifilis*.

The most striking feature shared by the three species of *Mariauxiella* is their uterine development, characterized by a ventral cortical uterine stem that gives rise to lateral diverticula bearing several dorsal outgrowths that cross the entire medulla and reach the dorsal cortex (de Chambrier & Rego, 1995; de Chambrier & Vaucher, 1999; present study). This pattern is similar to type 2 uterine development *sensu* de Chambrier et al. (2004), but differs by the presence of dorsal outgrowths extending from the ventral cortex to the dorsal cortex. It was described in detail by de Chambrier & Rego (1995) and designated as type 3 uterine development by Alves et al. (2021b). Apart from *Mariauxiella* spp., type 3 uterine development has been observed only in *Amazotaenia yvettae* de Chambrier, 2001, *Luciaella ivanovae* Gil de Pertierra, 2009, and *Man*. *piscatorum* (see de Chambrier & Vaucher, 1999; de Chambrier, 2001; Gil de Pertierra, 2009). These species, considering that *Man*. *piscatorum* was known as *Mar*. *piscatorum* until recently, were all assigned to the former subfamily Peltidocotylinae Woodland, 1934, together with *Jauella glandicephala* Rego & Pavanelli, 1985, and *Peltidocotyle* spp., all Neotropical catfish cestodes (Gil de Pertierra, 2009). Even though former peltidocotyline genera shared the cortical position of vitelline follicles and testes, and a medullary ovary with projections into the dorsal cortex (unfortunately not seen in our cross-sections), phylogenetic reconstructions have shown that most of these similarities represent examples of convergent evolution. This is a phenomenon commonly found across several proteocephalid lineages (de Chambrier et al., 2015a; Arredondo et al. 2017; Alves et al. 2021a, b).

The presence of a circular musculature in the suckers acting as sphincters is another relatively rare and independently inherited trait among proteocephalids (de Chambrier et al., 2009). Its distribution and appearance vary among taxa and can be used as a good discriminating character at species-level, yet with little systematic value (de Chambrier et al., 2009). Even though Alves et al. (2021b) diagnosed *Mariauxiella* species as having a circular musculature in the suckers forming a double crescent, i.e., two curved structures with their concave sides directed toward one another, only *Mar*. *pimelodi* exhibited this feature (see fig. 1 of de Chambrier & Rego, 1995). In contrast, *Mar*. *brevifilis* and *Mariauxiella saltogrande* **n. sp**. have a well-developed circular musculature at upper and lower marginal regions only.

Unlike uterine development and the presence of circular musculature in suckers, which are shared with other proteocephalids, the morphology of the distal vaginal canal in species of *Mariauxiella* appears to be unique to this genus. This structure is characterized by a symmetrically enlarged, chamber-like terminal portion that abruptly narrows at the level of the vaginal sphincter (de Chambrier & Rego, 1995; de Chambrier & Vaucher, 1999; present study). To the best of our knowledge, *Frezella vaucheri* Alves, de Chambrier, Scholz & Luque, 2015 is the only other proteocephalid that possesses a chamber-like enlargement of the terminal portion of the vaginal canal in most proglottides (Alves et al., 2015). However, in contrast to the symmetrical enlargement observed in *Mariauxiella* spp., the terminal vaginal canal of *F. vaucheri* is asymmetrically expanded.

The increasing availability of molecular data for proteocephalid tapeworms has made it possible to generate robust phylogenetic reconstructions and improve our understanding of their evolutionary interrelationships. However, deep branches within the so-called “Neotropical fish superclade” or “Clade VIII” remain largely unresolved (de Chambrier et al., 2015a; Alves et al., 2025, 2026). Likewise, although our results strongly support the monophyly of *Mariauxiella* and the sister relationship between the new species and *Mar. brevifilis*, which together form the sister lineage to *Mar. pimelodi*, their phylogenetic affinities with other Neotropical proteocephalids remain unclear due to low statistical support for most internal branches. MT-CO1 nucleotide divergence among species of *Mariauxiella* (7.8–15.1%) falls within the range of interspecific variation reported for Neotropical fish proteocephalids, as demonstrated by the well-characterized genus *Pseudoendorchis*, which exhibits interspecific divergences of 3.4–16.2% (Alves et al., 2021a).

Most proteocephalids have a strict (oioxenous) host specificity, i.e., are known only from a single fish host species (Scholz & Kuchta, 2017). This pattern is also observed in *Mariauxiella*, as *Mar*. *pimelodi*, *Mar*. *brevifilis*, and *Mariauxiella saltogrande* **n. sp.** are only found in *Pimelodus ornatus* (the unidentified *Pimelodus* sp. in the original description most likely corresponds to *P*. *ornatus*), *A*. *inermis*, and *A*. *militaris*, respectively. Gil de Pertierra (2009) reported *Mar*. *brevifilis* (as *Ageneiella brevifilis*) from *A*. *militaris* in Argentina, but this record may represent a misidentification of *Mariauxiella saltogrande* **n. sp.** given the morphological similarities between these two species. Additional sampling of auchenipterid catfishes, combined with robust taxonomic assessments, may reveal a higher diversity of *Mariauxiella* and closely related taxa; only three out of the 13 valid species of *Ageneiosus* have been scrutinized for tapeworms, with *A*. *inermis* alone harbouring as many as four species (Alves et al., 2017a). One of these species, *Luciaella ivanovae*, may be closely related to *Mariauxiella* due to their morphological similarities (Gil de Pertierra, 2009; Alves et al., 2021b) but it has yet to be molecularly characterized and subjected to phylogenetic analyses.

The three known species of *Mariauxiella* have been described from the Paraná River basin. However, *Mar*. *pimelodi* ex *P*. *ornatus* and an unidentified species of *Mariauxiella* ex *A*. *inermis* (reported as *Ageneiella* sp.) have also been recorded in the Amazon River basin (de Chambrier et al., 2015b; Alves et al., 2021a). Unlike *A*. *militaris*, which is restricted to the Paraná River basin, most species of *Ageneiosus* are found exclusively in the Amazon River basin (Ribeiro et al., 2017). Given the strict specificity exhibited by species of *Mariauxiella* and their apparent tight association with *Ageneiosus* spp., we hypothesize that the diversity of *Mariauxiella* in the Amazonia is likely higher than currently recognized and may exceed that observed in the Paraná River basin, thereby reflecting the greater host diversity in the former drainage.

## Supplementary Information

Below is the link to the electronic supplementary material.

Supplementary file1 (TXT 133 kb)

Supplementary file2 (TXT 150 kb)

Supplementary file3 (TXT 281 kb)

Supplementary file4 (TXT 12 kb)

Supplementary file5 (TXT 12 kb)

Supplementary file6 (TXT 41 kb)

## Data Availability

Type specimens were deposited in the Helminthological Collection of the Instituto Oswaldo Cruz, Rio de Janeiro, Brazil. Newly generated sequences were deposited in the GenBank database, and alignments and phylogenetic trees are available in the Online Resources.
